# Hyaluronan (HA) Interacting Proteins RHAMM and Hyaluronidase Impact Prostate Cancer Cell Behavior and Invadopodia Formation in 3D HA-Based Hydrogels

**DOI:** 10.1371/journal.pone.0050075

**Published:** 2012-11-16

**Authors:** Lisa A. Gurski, Xian Xu, Lyana N. Labrada, Ngoc T. Nguyen, Longxi Xiao, Kenneth L. van Golen, Xinqiao Jia, Mary C. Farach-Carson

**Affiliations:** 1 Department of Biological Sciences, University of Delaware, Newark, Delaware, United States of America; 2 Department of Materials Science and Engineering, University of Delaware, Newark, Delaware, United States of America; 3 The Center for Translational Cancer Research, University of Delaware, Newark, Delaware, United States of America; 4 Department of Biochemistry and Cell Biology, Rice University, Houston, Texas, United States of America; 5 Department of Bioengineering, Rice University, Houston, Texas, United States of America; Johns Hopkins University, United States of America

## Abstract

To study the individual functions of hyaluronan interacting proteins in prostate cancer (PCa) motility through connective tissues, we developed a novel three-dimensional (3D) hyaluronic acid (HA) hydrogel assay that provides a flexible, quantifiable, and physiologically relevant alternative to current methods. Invasion in this system reflects the prevalence of HA in connective tissues and its role in the promotion of cancer cell motility and tissue invasion, making the system ideal to study invasion through bone marrow or other HA-rich connective tissues. The bio-compatible cross-linking process we used allows for direct encapsulation of cancer cells within the gel where they adopt a distinct, cluster-like morphology. Metastatic PCa cells in these hydrogels develop fingerlike structures, “invadopodia”, consistent with their invasive properties. The number of invadopodia, as well as cluster size, shape, and convergence, can provide a quantifiable measure of invasive potential. Among candidate hyaluronan interacting proteins that could be responsible for the behavior we observed, we found that culture in the HA hydrogel triggers invasive PCa cells to differentially express and localize receptor for hyaluronan mediated motility (RHAMM)/CD168 which, in the absence of CD44, appears to contribute to PCa motility and invasion by interacting with the HA hydrogel components. PCa cell invasion through the HA hydrogel also was found to depend on the activity of hyaluronidases. Studies shown here reveal that while hyaluronidase activity is necessary for invadopodia and inter-connecting cluster formation, activity alone is not sufficient for acquisition of invasiveness to occur. We therefore suggest that development of invasive behavior in 3D HA-based systems requires development of additional cellular features, such as activation of motility associated pathways that regulate formation of invadopodia. Thus, we report development of a 3D system amenable to dissection of biological processes associated with cancer cell motility through HA-rich connective tissues.

## Introduction

A majority of patients who die from solid tumors each year suffer from bone metastases [1]. The three most commonly diagnosed cancer types, prostate, lung, and breast, metastasize to bone. Bone metastasis dramatically lowers quality of life due to pain, fractures, and hypercalcemia [2]. Additionally, presence of bone metastases lowers survival rates. For prostate cancer (PCa), the five year survival rate for local disease is 100% while the rate drops to 30% for advanced disease with distant metastases [1]. Currently, there are few effective therapies to treat bone metastasis or to prevent metastasis to bone or other sites [2]. A defined clinical need thus exists to develop new therapies to treat and prevent bone metastasis involving motile, invasive cancer cells that can readily colonize connective tissues such as bone marrow.

The study of pathways that control cancer cell invasion or migration, and the development of new drug to prevent these processes requires systems that can measure the invasive properties of these cells [3], [4]. Currently available invasion and migration assays are less than ideal in that they often: 1) are difficult to quantify, 2) are difficult to tailor to individual cancer cell behaviors, or 3) employ matrices such as animal derived Matrigel™ that contain growth factors that complicate experimental results [3], [5], [6].

The bone marrow matrix consists of soft, gel-like connective tissue rich in hyaluronic acid (HA) [7], [8]. HA is a large, non-sulfated glycosaminoglycan comprised of repeating β-1,4-linked D-glucuronic acid and β-1,3 N-acetyl-D-glucosamine disaccharide units [9]. HA is found ubiquitously in the extracellular matrix (ECM) of all cell types, but is particularly enriched in connective tissues. Cells can bind HA through various receptors, including cluster of differentiation 44 (CD44) or receptor for hyaluronan-mediated motility (RHAMM) [10]. In cancer cells, RHAMM has been shown to bind CD44 on the cell surface, and HA binding to this complex promotes downstream signaling resulting in Rho GTPase activation and increased cell migration [11], [12]. Both RHAMM and CD44 expression levels have been linked to progression of a number of cancers, including PCa [13], [14].

Another way that cells interact with HA is by degrading it, by expression and secretion of hyaluronidases (HAases). Relevant to PCa invasion are the HAases, Hyal-1 and Hyal-2, whose expression levels have been implicated in PCa metastasis [13], [15], [16]. Hyal-1 is active at low pHs from 3.5–3.8 and cleaves any molecular weight HA into tetramers [17]. Hyal-2 shows optimal activity at pH 6.0–7.0 and cleaves high molecular weight HA into intermediate, 20 kDa size fragments [18]. A glycosylated, 57 kDa form of Hyal-1 can be secreted by cells [19]. HAase secretion may facilitate PCa metastasis by allowing cancer cells to invade the HA-rich connective tissue matrix.

Importantly, HAases are druggable targets, indicating their potential use as targets to slow invasion and possibly prevent metastasis [20], [21]. One HAase inhibitor, disodium cromoglycate (DSC) [22], [23] is FDA approved for use to relieve symptoms of allergy and asthma, and shows low toxicity in patients [24], [25]. Its ability to inhibit PCa invasion and metastasis has not been investigated.

We previously described the development of a HA-based hydrogel for culture of poorly adherent bone metastatic PCa cells [26]. The hydrogel consists of two chemically modified HA components, aldehyde-carrying HA (HAALD) and adipic acid dihydrazide-derived HA (HAADH). These components crosslink via the formation of hydrazine linkages, releasing water as the only byproduct [27]. PCa cells can be encapsulated in the HA hydrogel during the crosslinking process. Viability of the encapsulated cells remains high for at least a week [26]. Because the HA is of bacterial origin, it is devoid of eukaryotic growth factors that could affect cell growth, invasion, and migration.

To study the development of invasive properties of PCa cells in a native connective tissue matrix, we used the LNCaP series of human PCa cell sublines that were developed to mimic the process of PCa bone metastasis. These cells are regarded as one of the better PCa progression models available because of their ability to form clinically relevant osteoblastic lesions in mice [28]. Individual sublines in the LNCaP series reflect the steps in disease progression with LNCaP cells representing an early prostate tumor that has reached a nearby lymph node, C4 cells representing aggressive, locally invasive cancer cells that survive after castration, C4-2 cells representing aggressive, metastatic disease, and C4-2B cells representing PCa castration resistant bone metastases [29]. Using these cells and the novel 3D HA gel invasion system we developed, we investigated the role of HA receptors and HAases in PCa invasion and migration through an HA-rich connective tissue like matrix. For the purposes of this work describing the movement of PCa cells in 3D hydrogels, which differs from migration across plastic surfaces, we describe migration as the apparent movement of cells through porous hydrogels evidenced by extension of cellular processes we call “invadopodia” and by the merging of formerly separated clusters in the hydrogels.

## Materials and Methods

### Materials

Electrophoresis, cell culture, and transfection reagents and supplies were purchased from Invitrogen (Carlsbad, CA). TCM™ defined serum replacement was purchased from MP Biomedicals (Solon, OH). Hyaluronic acid (sodium salt, 500 KDa and 1.3 MDa) was generously donated by Genzyme Corporation (Cambridge, MA). RHAMM (HMMR) and CD44 antibodies were purchased from Novus Biologicals (Littleton, CO). Hyal2 and β-actin antibody were purchased from Abcam (Cambridge, MA). Equine-α-mouse-HRP secondary antibody was purchased from Cell Signaling Technology (Danvers, MA). Hyal1 antibody, bovine testicular hyaluronidase (BTH), β-mercaptoethanol (βME), glycine, Tween®20, bovine serum albumin (BSA), sodium formate, Coomassie brilliant blue, disodium cromoglycate (DSC), and deoxycholic acid were purchased from Sigma Aldrich (St. Louis, MO). Goat-α-rabbit-HRP secondary antibody, protease inhibitors (PI), 5X sample buffer, sulfo-NHS-SS-biotin, and avidin agarose were purchased from Thermo Scientific (Rockford, IL). Laemmli sample buffer and alcian blue were purchased from Biorad (Richmond, CA). Tris buffer, glacial acetic acid, methanol, low melting point (LMP) agar, dimethyl sulfoxide (DMSO), sodium dodecyl sulfate (SDS), Triton X-100, and sodium chloride (NaCl) were purchased from Fisher Scientific (Fair Lawn, NJ). Nonidet P-40 was purchased from Roche (Indianapolis, IL) and ethanol was purchased from Decon Labs, Inc (King of Prussia, PA). All compounds used were reagent grade or better.

### Cell Culture

Low passage number PCa cells were maintained in Corning (Lowell, MA) tissue culture 75 mm flasks at 37°C in 5.0% (v/v) CO_2_ in T-medium supplemented with 5% (v/v) fetal bovine serum (FBS) and 100 U/ml penicillin G sodium and 100 µg/ml streptomycin sulfate in 0.085% (w/v) saline (PS). Medium was changed every 2–3 days. Cells were passaged at approximately 80% confluency, judged by eye, using 0.25% (w/v) trypsin with ethylenediaminetetraacetic acid (EDTA) 4•Na. LNCaP sublines [30] were generously provided by Dr. Leland Chung and PC3 cells were purchased from ATCC (Manassas, VA).

### Preparation of HAALD and HAADH

To synthesize the HA aldehyde (HAALD), oxidation reaction of HA (1.3 MDa) was performed in the presence of sodium periodate under aqueous conditions. Adipic dihydrazide (ADH)-modified HA (HAADH) was synthesized by carbodiimide-mediated coupling of ADH with the carboxyl groups of HA (500 KDa) in aqueous conditions. Detailed procedures for the synthesis and characterization of both of these HA derivatives was reported in our previous study [26].

### Cell Culture in 2D and 3D Conditions

HAALD and HAADH were dissolved separately in Dulbecco’s phosphate buffered saline (DPBS) to a concentration of 10 mg/mL overnight at 37°C. 2% (w/v) LMP agar in DPBS was prepared and was melted in a 70°C heatblock (Labnet International, Woodbridge, NJ) at least 1 hour prior to use. Dissolved HA derivates were sterilized by UV irradiation at 254 nm for 15 minutes prior to use.

PCa cells were released from their flask using 0.25% (w/v) Trypsin EDTA 4NA and the total number of cells was counted using a hemocytometer (Hausser Scientific, Horsham, PA). 100,000 (for 24-well plate) or 600,000 (for 6-well plate) cells were pelleted for each culture. Cell culture inserts (Millipore, Billerica, MA, pore size: 0.4 mm, diameter 12 mm for 24-well plate, 30 mm for 6 well plate) were pre-wet in T-medium then placed in the wells of a 24-well or 6-well culture plate (Beckton–Dickenson Labware, Franklin Lakes, NJ). For 2D cultures, cell pellet was mixed with T-medium (1 mL for 24-well plate, 4 mL for 6-well plate) and plated.

For 3D HA hydrogel culture, cell pellet was mixed with 100 µl (for 24-well plate) or 300 µl (for 6-well plate) of HAALD. An equal volume of HAADH was added and the culture was mixed well to evenly disperse cells. The hydrogel solution then was pipetted into pre-wet cell culture inserts and allowed to solidify for approximately 10 minutes at 37°C. T-medium (1mL for 24-well plate, 4 mL for 6-well plate) supplemented with 1% (v/v) PS and either 5% (v/v) FBS or 2% (v/v) TCM™ was added around the insert and the culture was incubated at 37°C, 5% (v/v) CO_2_. This concentration of TCM™ is consistent with previous studies supplementing C4-2 culture [31], [32].

For 3D agar culture, cell pellet was mixed with 85 µl (for 24-well plate) or 510 µl (for 6-well plate) DPBS warmed to 37°C. Pre-melted 2% (w/v) LMP agar was added to DPBS/cell mixture to a final concentration of 0.3% (w/v) LMP agar. Agar culture was incubated at room temperature for approximately 5 minutes before pipetting into the cell culture insert to prevent agar gel from leaking through the membrane pores. Once plated in insert, the agar gel was allowed to solidify for approximately 10 minutes at room temperature. Solidification was performed at room temperature to expedite gelation of soft agar. T-medium (1 mL for 24-well plate, 4 mL for 6-well plate) was subsequently added around the insert and the culture was incubated at 37°C, 5% (v/v) CO_2_. For all cultures, half medium changes were performed every two days.

Metabolic activity and cell counts were performed for the cell types and culture conditions described. To measure metabolic activity of cells, water soluble tetrazolium salt-1 (WST-1, Roche) reagent was utilized as described [33] with the following modifications: the assay was performed in a 24 well plate on cells grown for either three or six days. WST-1 reagent (100 µl) was applied to 1 mL culture medium and the plate was incubated at 37°C for 1 hour. After incubation, 100 µl of culture medium was removed and placed in a 96 well plate. Absorbance was measured at 450 nm in a DTX880 multimode detector (Beckman Coulter, Brea, CA). To count cell numbers within the HA hydrogel, phase contrast images were utilized. Within these images, an average-sized cell cluster with clear cell boundaries was selected and the number of cells in the cluster was counted. This number was multiplied by the total number of clusters in the image, yielding an approximate total cell count per photographed field.

### Invasion Assay Quantification Methods

For all invasion assays, phase contrast images were taken of each cell culture using a Nikon Eclipse TE2000-U (Tokyo, Japan) microscope and a 10X objective. The average number of invadopodia was determined by counting the total number of invadopodia per photographed field for three biological replicates. An “invadopodium” was defined as a thin cellular process extending outward from a cell cluster, and clear enough to be easily identified by eye. The percentage of merging clusters was calculated by counting both the number of clusters in physical contact with another cluster and the number of free clusters for three biological replicates. From these values, the percentage of merging clusters was calculated and considered an index of migration in 3D hydrogels. In samples where networks of cell clusters were observed, a single cell cluster was defined as a distinct, rounded area of the cellular network sometimes appearing to contain a higher cell density. For both types of quantification, care was taken to ensure consistency when counts were being performed.

### Rheology

Rheological characterization of hydrogel samples were performed on a stress-controlled rheometer (AR-G2, TA Instruments, New Castle, DE) with a 20 mm diameter standard steel parallel-plate geometry and at a 100 µm gap. Dynamic oscillatory time sweeps were performed at 25°C or 37°C for agar and HA gels respectively, and the storage (G‘) and loss (G“) moduli were recorded. 30 µl agar solution (0.3% w/v) or HAADH/HAALD mixture (1% w/v) was loaded into the geometry that was subsequently covered with mineral oil at the edge to prevent water evaporation. These mixtures were allowed to solidify *in situ* as the measurements were taken. Dynamic oscillatory time sweeps were collected at angular frequencies of 6 rad/s and 1% strain chosen from the linear viscoelastic regime [34]. These experiments were repeated on at least three samples and averaged data are presented.

### Protein Extraction

The cells combined from two wells of a 6-well plate culture were used for protein extraction experiments. BTH (180 µl at 10 mg/mL) was applied and cultures were incubated at 37°C for 30 minutes. The cultures were transferred to centrifuge tubes and cells were pelleted by centrifugation for 1 minute at 3000 RPM. Cells were washed once with DPBS. Radioimmunoprecipitation (RIPA) buffer (50 mM Tris buffer pH 8.0, 150 mM NaCl, 0.5% (w/v) deoxycholic acid, 1% (v/v) Nonidet P-40, 0.1% (w/v) SDS, ddH_2_O, 200 µl) containing PI was applied to the cell pellets. Lysates were incubated on ice for 45 minutes with occasional vortexing. Lysates were cleared by centrifugation at 13,000 rotations per minute (RPM) for 10 minutes. The total protein concentration from the lysates was determined using a bicinchoninic acid (BCA) protein assay (Thermo Scientific) following a standard protocol. Lysates were stored at −20°C.

### Western Blotting

Protein (50 µg) was mixed with RIPA buffer and 5X Lane Marker Sample Buffer and βME (3% (v/v) final) to a total volume of 25 µl. Samples were boiled for 5 minutes, then cooled and quickly spun. Samples and a protein ladder were electrophoresed on a NUPAGE 4–12% Bis-Tris gel using an X-Cell Surelock™ electrophoresis cell and MOPS running buffer. Samples were transferred to a nitrocellulose membrane using an X-Cell™ II transfer apparatus and a transfer buffer consisting of 1.5125 mg/mL Tris, 7.2 mg/mL glycine, and 0.01% (w/v) SDS at a setting of 21V for 1.5 hours.

The membrane was blocked in 3% (w/v) BSA in DPBS containing 0.1% Tween® 20 (PBST) for 1 hour at room temperature with gentle shaking. Primary antibodies (1∶10,000 for RHAMM, 1∶5000 for CD44 and β-actin, 1∶500 for Hyal1 and Hyal2) in 3% (w/v) BSA in PBST were incubated with the membrane for 2.5 hours at room temperature with shaking. The membrane was washed 3 times, 10 minutes each time, with PBST. Secondary antibodies (1∶5000 goat-α-rabbit HRP or 1∶10,000 equine-α-mouse HRP) in 3% (w/v) BSA (for Hyal1, Hyal2, and β-actin blots) or 3% (w/v) non-fat dried milk (for RHAMM and CD44 blots) were incubated with the membrane for 1 hour at room temperature with shaking. The membrane was washed 3 times, 10 minutes each time, with PBST. Supersignal® West Dura Extended Duration Substrate (Thermo Scientific) was prepared as directed and applied to the membrane for 5 minutes at room temperature with shaking. The blot was exposed using BioMax Light Film (Kodak, Rochester, NY) and developed in an SRX-101A developer (Konica Minolta Medical & Graphic Inc, Tokyo, Japan). PC-3 cell lysate (50 µg) was used as a positive control for CD44 westerns.

### Immunostaining Protocol

Cells grown on 2D were cultured in an 8 well Lab-Tek II Chambered Coverglass (Nalge Nunc, Naperville, IL). The medium was removed and chambers were washed 2 times with DPBS. Cells were fixed for 10 minutes in 4% (v/v) paraformaldehyde (PFA) (Electron Microscopy Sciences, Hatfield, PA) in ddH2O. Excess PFA was removed and the chambers were washed twice with DPBS. Triton X-100 in DPBS (0.2% (v/v)) was prepared and applied to chambers for 10 minutes. Excess Triton X-100 solution was removed and chambers were washed twice with DPBS. Cultures were blocked in 3% (w/v) BSA in DPBS at 4°C overnight. Cells were incubated with 1∶1000 (v/v) solution of RHAMM or CD44 primary antibodies in 3% BSA at 4°C overnight. A 1∶1000 (v/v) solution of Draq5 (Biostatus Limited, Leicestershire, UK) in DPBS was applied for 10 minutes at room temperature. Chambers again were washed with DPBS and Gel/Mount™ (Biomeda, Foster City, CA) was added to chambers to prevent photobleaching. Cells were visualized using confocal microscopy on a Zeiss LSM 510 VIS (Carl Zeiss, Maple Grove, MN).

Cells in 3D were cultured in 24-well plates as described above. Cell clusters were gently removed from the hydrogel using a micropipette. Clusters were washed gently with 1 mL DPBS 2 times, quickly centrifuging to collect cell clusters each time. Clusters were carefully resuspended in 4% (v/v) PFA and transferred to an 8 well chambered coverglass. Cultures were incubated at room temperature for 10 minutes. Once transferred to the chambered coverglass, the same staining procedures were used as employed for 2D culture. Extreme care was taken to retain as many cell clusters as possible during each liquid removal step.

### Cell Surface Biotinylation Assay

C4-2 cells were cultured in a T-75 flask to approximately 90% confluency. Cells were released from the flask, suspended in T-medium and counted. Cells were washed three times in ice-cold PBS (pH 8.0, 5 mL). Cells were resuspended in ice-cold PBS (pH 8.0) to a final concentration of 20×10^6^ cells/mL. Freshly prepared, 10 mM sulfo-NHS-SS-biotin (80 µl) was added to the cell mixture and the mixture was incubated at room temperature for 30 minutes with shaking. 50 mM Tris (pH 8.0, 2 mL) was added to deactivate the biotinylation reaction, and the cell pellet was subsequently washed twice with PBS (pH 8.0, 2 mL). The final PBS wash was removed completely from the cell pellet, and RIPA buffer containing PI (500 µl) was applied to the cell pellet. The lysis reaction was incubated on ice for one hour. The lysate was cleared by centrifuging at 12,000 RPM for 10 minutes, after which the supernatant was removed and stored at –20°C.

A BCA assay was performed on the biotinylated lysate as described previously; approximately 1.5 mg of protein was obtained. Avidin agarose (1 mL) was applied to a microcentrifuge tube and the resin was settled by centrifuging 1 minute at 2000 RPM. The resin was washed three times with RIPA buffer (500 µl). The lysate was applied to the resin and the mixture was incubated on a tube rotator at 4°C overnight. The resin was settled by centrifuging 1 minute at 2000 RPM and the unbound lysate was removed. Unbound lysate (10 µl) was mixed with Laemmli Sample Buffer containing 5% (v/v) βME (10 µl). The resin was washed three times with RIPA containing PI (500 µl). Laemmli Sample Buffer containing 5% βME (50 µl) was applied to the beads. Laemmli-containing mixtures were boiled for 5 minutes, then spun down at 13,000 RPM. The biotinylated pulldown and unbound fraction were electrophoresed and transferred to nitrocellulose as described previously. Bone metastatic PCa cell (PC3) lysate (10 µl) mixed with Laemmli sample buffer containing βME (10 µl) was included as a positive control for CD44 expression. Western blotting for CD44, RHAMM, and GAPDH were performed as described under the “Western Blotting” section.

### RHAMM Knockdown

C4-2 cells were maintained in antibiotic-free medium for three days prior to transfection. Mixed 6 pmol/cm^2^ Stealth RNAi™ siRHAMM duplex (GGCGUCUCCUCUAUGAAGAACUAUA and UAUAGUUCUUCAUAGAGGAGACGCC) and 0.5 µl/cm^2^ Lipofectamine™ RNAiMAX in Optimem® I for RHAMM knockdown or 6 pmol/cm^2^ low GC Stealth RNAi™ negative control duplex and 0.5 µl/cm^2^ Lipofectamine in Optimem for transfection control. Transfection mixtures were incubated for 15 minutes at room temperature before applying to cells. Transfection reactions were incubated for six hours at 37°C and plates were swirled gently every hour to mix transfection reagents.

The transfection mixture was aspirated from the cells, and cells were washed with DPBS. Antibiotic free medium was applied to the cells and transfected cells were incubated at 37°C overnight before using for experiments. Efficacy of RHAMM knockdown was assessed through Western blotting for RHAMM as described under the “Western Blotting” section.

### Hyaluronidase Activity Assay

HAase Activity was examined via substrate gel electrophoresis [35], [36]. Low molecular weight HA was dissolved overnight at 37°C in distilled water (0.17 mg/mL). This HA solution was used in lieu of water in the preparation of a 12% (w/v) ProtoGel® acrylamide gel (National Diagnostics, Cherry Hill, NJ). LNCaP, C4, C4-2 and C4-2B lysates or 50 µg BTH were mixed with equal volumes of Lamelli sample buffer (5% (v/v)) and loaded on the HA-containing gel. The gel was electrophoresed using an X-Cell Surelock™ electrophoresis cell and MOPS running buffer. The gel then was removed from the cassette and washed for one hour at room temperature in 3% (v/v) Triton X-100 in PBS. The gel then was incubated in a formate-NaCl buffer (0.1 M Sodium Formate and 0.15 M sodium chloride dissolved in distilled water, pH 4.6) for 16–20 hours at 37°C. The gel was washed twice with distilled water then incubated with a 0.5% (w/v) Alcian Blue in 3% (v/v) glacial acetic acid solution for one hour to detect the presence of HA. The gel was washed three times for one hour each with a 7% (v/v) acetic acid solution for destaining and fixation. The gel then was washed twice with distilled water, and twice with a 50% (v/v) methanol, 10% (v/v) glacial acetic acid solution. To detect the presence of protein on the gel, it was then incubated with a 0.25% (w/v) Coomassie brilliant blue in 9% (v/v) ethanol, 45.5% (v/v) glacial acetic acid for one hour. This was followed by three one hour washes with the methanol/acetic acid washing solution.

### Treating Cultures with HAase Inhibitor

A solution of 125 mM DSC was prepared in 40% (v/v) DMSO and filtered in a Steriflip filter (Millipore) to sterilize. DSC (3.33 or 10 µl of 125 mM) was added to 800 µl or 2.4 mL T-medium in 24 or 6 well plates, respectively, to a final concentration of 500 µM DSC, the IC-50 value of this inhibitor [37]. An equal amount of 40% (v/v) DMSO was used as a vehicle control. Medium was changed every three days and fresh DSC or DMSO solution was applied as described.

### Trypan Blue Exclusion

The toxicity of DSC on PCa cells was determined using a trypan blue exclusion assay. 600,000 cells were plated into 6 well plates with T medium, 5% (v/v) FBS, 1% (v/v) PS (4 mL). After 24 hours of culture, the cultures were treated with DSC or vehicle control as described previously. At three and six day timepoints, medium was aspirated and cells were released with trypsin (250 µl). Cells were pelleted, then resuspended in 500 uL T medium. 50 uL of a 0.4% (w/v) solution of trypan blue in buffered isotonic salt solution was added to each sample. The total number of white (live) and blue (dead) cells was determined by counting on a hemocytometer. The percentage of live cells was determined from this data.

### Statistical Analysis

Error bars on all figures display standard error of the mean (SEM). Significance was determined using Student’s two sample two-tailed t-tests with p<0.05 considered significant.

## Results

### Invadopodia and Cluster Formation in 3D HA Hydrogels

#### Invadopodia and cluster formation response to motogenic factors

In initial experiments, we evaluated C4-2 cell morphology in the HA hydrogel cell culture system to determine if differences could be seen in response to FBS used as a motogenic factor to stimulate motility. Control cultures were treated with TCM™, a plant-based serum replacement to maintain cell growth and viability, without the ability to promote invasion and migration. While TCM™ treated C4-2 cells showed a lower metabolic output compared to FBS, assessed by WST assay, there was no significant difference in estimated cell counts for cells growing in either condition at either three or six day timepoints (see Table S1). We observed and quantified morphology differences of cells in these gels as a measure of cell invasion potential and migration ability.

**Figure 1:**
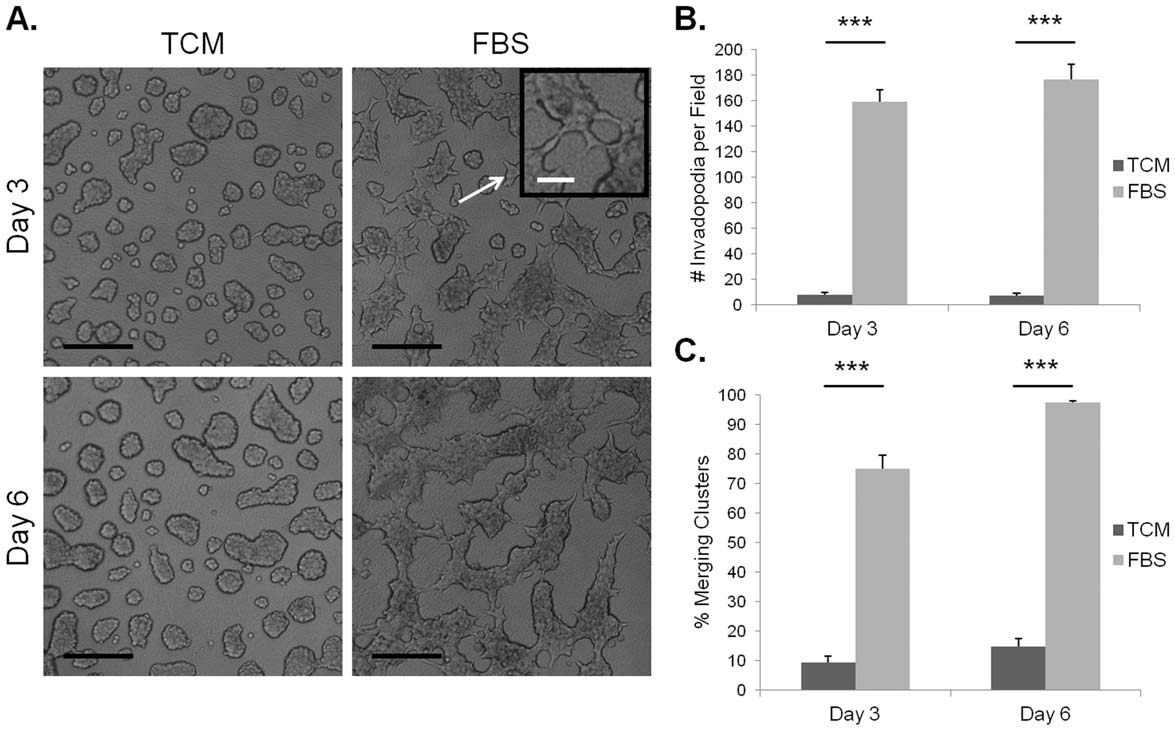
Invadopodia and cluster merging in the HA hydrogel invasion assay depend on treatment with motogenic factors. Images of C4-2 cells cultured for three or six days with either 2% TCM™ (control) or 5% FBS (motogenic factor) in the HA hydrogel invasion assay. Arrow and inset show a magnified view of the image to better display invadopodia. Black scale bars represent 200 µm, white scale bar on inset represents 50 µm (A). Quantification for average number of invadopodia (B) or percent merging clusters (C) in each imaged field. Error bars = SEM, n = 3, ***p<0.001.

As shown in Figure 1A, at both three and six day timepoints, the TCM™ treated C4-2 cultures showed smaller, well-defined cell clusters mostly devoid of invasive cellular processes (invadopodia). In contrast, the FBS-treated cultures showed larger cell clusters that appeared more uneven in shape. Remarkably, the FBS treated clusters were regularly seen to be merging together and displayed a considerable number of clearly evident invadopodia at each timepoint. The inset of Figure 1A displays a magnified view of these invadopodia structures. The cell clusters appeared larger after six days than after three days for each treatment, but the number of invadopodia was relatively constant between the two timepoints.

Quantification of the number of invadopodia revealed that the number of these structures was significantly higher (p<0.0001) for FBS treatment than for TCM™ treatment at both three and six day timepoints (Figure 1B). The number of invadopodia did not increase for either treatment between the two timepoints. Quantification of merging cluster percentage showed that FBS treated C4-2 cell clusters were more likely to be merging compared to TCM™ treated cells for both three and six day timepoints (Figure 1C). Collectively, these results show that the HA hydrogel invasion assay can be utilized to test the effects of motogenic factors on PCa cell invasion. Importantly, because TCM™ supports cell growth, these data suggest that the processes of invadopodia formation and merging clusters are independent of cell division.

#### Invadopodia and cluster formation in a PCa progression model

Next, we evaluated the parameters of invadopodia number and merging cluster percentage in the HA hydrogel for PCa cells representing increasing disease progression. For this study we compared highly aggressive, metastatic C4-2 cells to their less aggressive LNCaP parent cell line. Similar metabolic activities and cell counts were observed when comparing LNCaP and C4-2 cultures at three and six day timepoints (see Table S1). We hypothesized that the differences in invadopodia number and/or merging cluster percentage observed in Figure 1 also would be present when comparing growth of LNCaP and C4-2 cells in the HA hydrogels.

**Figure 2:**
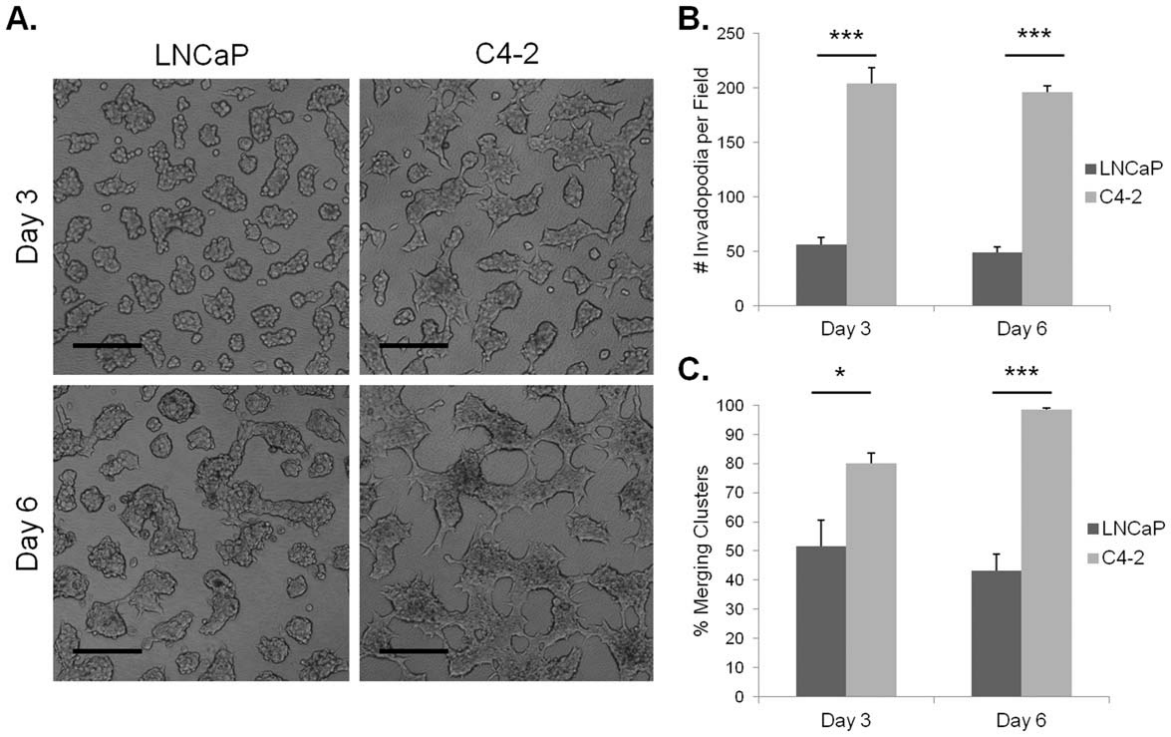
Invadopodia and cluster merging in the HA hydrogel invasion assay depend on invasive capability. Images of LNCaP or C4-2 cells cultured for three or six days with 5% FBS in the HA hydrogel invasion assay. Scale bars represent 200 µm (A). Quantification for average number of invadopodia (B) or percent merging clusters (C) for each imaged field. Error bars = SEM, n = 3, *p<0.05, ***p<0.001.

At both three and six days of culture in the HA hydrogel, LNCaP cells grew in well-defined, irregularly shaped, grape-like clusters (Figure 2A). Few invadopodia were observed projecting from the LNCaP cell clusters. C4-2 cells showed a similar morphology as described in Figure 1, growing in large, poorly-defined, merging clusters with abundant numbers of invadopodia. When quantified, the number of invadopodia was significantly different (p<0.001) between LNCaP and C4-2 cell culture at both timepoints (Figure 2B). The number of invadopodia did not differ between the three and six day timepoints for either of the cell lines. For both timepoints, C4-2 cultures showed a higher percentage of merging clusters compared to LNCaP, although the difference was more dramatic on day six than day three (Figure 2C). Therefore, the HA hydrogel invasion assay can be utilized to compare invasion among different cancer cell lines. Because LNCaP and C4-2 cells show similar rates of cell growth, but different levels of invadopodia and cluster convergence these results again demonstrate that these behaviors are independent of cell division.

### Comparison of HA Hydrogel to Soft Agar Gel

We suspected that the invadopodia and merging cluster formation observed in the HA hydrogels was due in part to the chemical make-up of the hydrogel, not simply its mechanical properties or the process of being encapsulated in 3D. To test this, we compared C4-2 cell growth in HA hydrogels to that in non-physiologically relevant soft agar gels.

**Figure 3:**
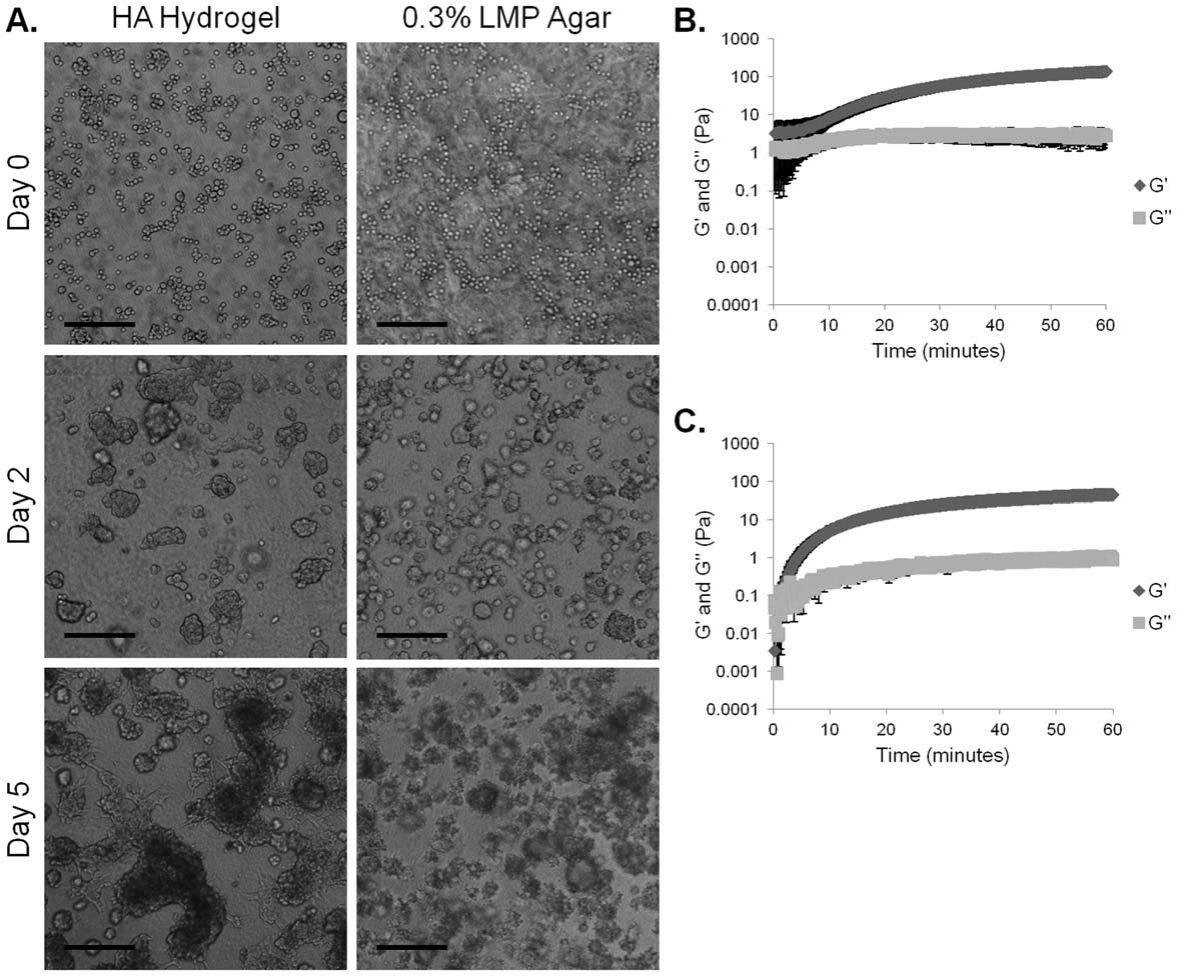
Cells cultured in the HA hydrogel show differences in morphology compared to an agar gel of matched stiffness. Images of C4-2 cells cultured for zero, two, or five days with 5% FBS in the HA hydrogel or in 0.3% LMP agar. Scale bars represent 200 µm (A). Average elastic (G’) and loss (G”) moduli across one hour time for HA hydrogel (B) or 0.3% LMP agar (C). Error bars = SEM, n = 3.

The rheological properties of the HA hydrogels were compared to that of 0.3% agar in Figure 3 (B and C). At time zero, the HA hydrogels had a higher G’ than G”, suggesting that gelation occurred once HAADH and HAALD were mixed. The elastic modulus continued to increase, reaching a plateau value of 139±21 Pa at 1 h. Conversely, the agar solution gelled to reach a final elastic modulus of 46±6 Pa. While the elastic modulus provides a measure of gel stiffness, tan(δ), defined as G”/G’, is a useful indicator of hydrogel damping properties [38]. Here, the final tan(δ) values for HA gels and the agar gels were similar (0.02±0.01 and 0.02±0.00, respectively) and close to zero, characteristic of elastic hydrogels. Overall, HA and agar gels had similar dissipative properties although the former was slightly stiffer than the latter.

In soft agar, C4-2 cells formed small cell clusters that appeared to grow in size slightly between two and five days of culture (Figure 3A). However, compared to HA hydrogel cultures, cells in the soft agar formed smaller clusters. Merging clusters that send out invadopodia were not observed. Therefore, the ability of the cells to form these merging clusters and invadopodia appeared to be prominent only when HA was used as the 3D matrix.

While both HA and agar gels are soft and elastic, it is important to note that these gels differ in that the HA gel crosslinks covalently while the agar gel crosslinks physically. The crosslinking method, along with possible differences in porosity and stiffness, represent additional variables between the two gels. While we cannot know if any of these variables may account in part for the observed cell behavior, it is clear that the HA gels provide context-specific signals to the cells that the agar gels do not.

### HA Receptor Expression in PCa Cells

**Figure 4:**
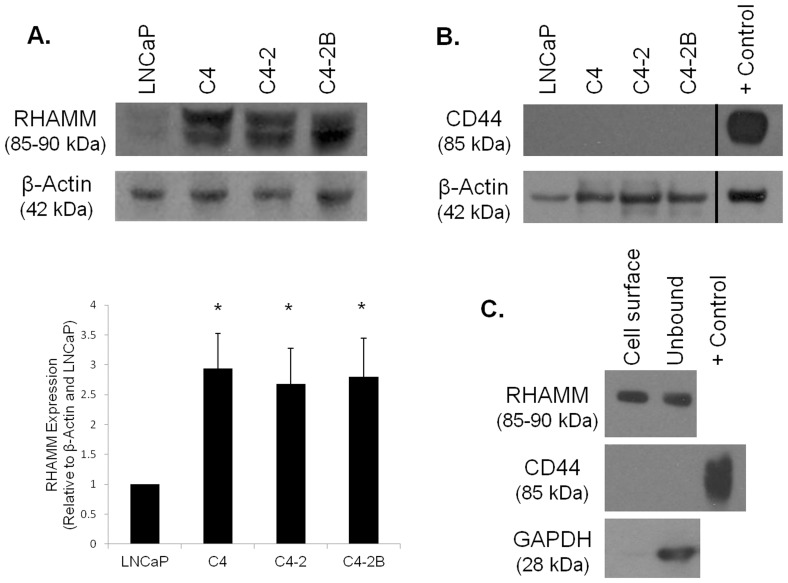
Cells of the LNCaP series express cell-surface RHAMM in the absence of CD44. Western blotting and densitometry for RHAMM (A) and western blotting for CD44 (B) across the LNCaP series of cell lines with β-actin used as a load control. Cell surface biotinylation for RHAMM and CD44 with GAPDH utilized as a cytoplasmic control (C). PC-3 lysate was used as a positive control for CD44, Error bars = SEM, n = 3, *p<0.05 compared to LNCaP.

To understand how PCa cells interact with the HA hydrogel to form merging clusters and invadopodia within the invasion assay, we investigated the expression of several classes of HA-interacting proteins, starting with the HA receptors. Western blotting showed that both common isoforms of RHAMM were expressed across the LNCaP series, with lower expression by LNCaP cells compared to the more aggressive cell lines (Figure 4A). CD44 expression was not detected in the LNCaP cell series (Figure 4B) although it was expressed in the PC3 cell line used as a positive control.

Because RHAMM is reportedly found to be bound to CD44 on the cell surface, we were interested to see if RHAMM was present on the cell surface where it could interact with the HA hydrogel in the absence of CD44. To test this, we performed a cell-surface biotinylation assay under non-permeabilizing conditions where only proteins accessible on the cell surface would be labeled (Figure 4C). The presence of RHAMM, but not CD44, in the biotinylated, cell surface samples showed convincingly that RHAMM was bound to the cell surface of C4-2 cells even in the absence of CD44, and indicates that CD44 is not required for cells to express RHAMM on their surfaces where it can engage HA. GAPDH was used as a cytoplasmic control and its lack of labeling confirmed that the cells used in these experiments were not permeabilized and further that the column was washed sufficiently, removing all traces of unbiotinylated, cytoplasmic proteins.

### RHAMM Expression and Localization in 3D vs. 2D

**Figure 5:**
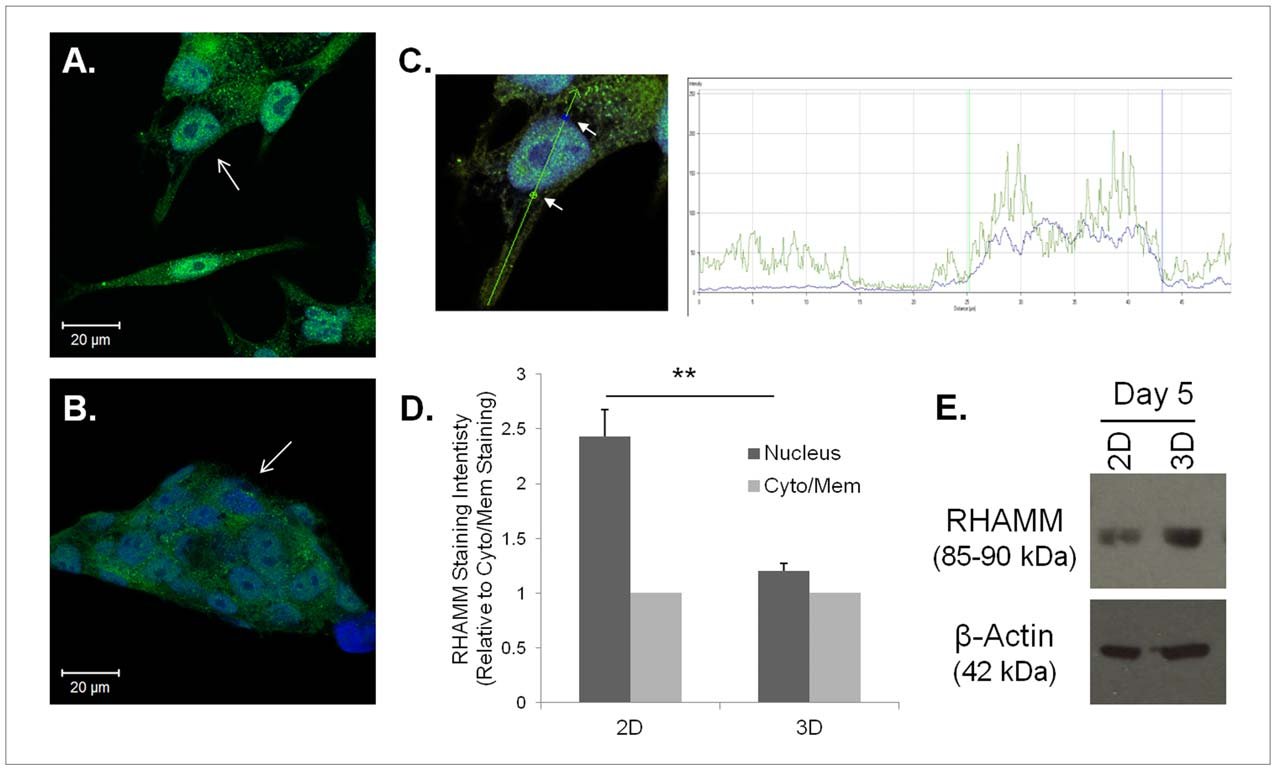
HA hydrogel culture changes expression and localizes RHAMM to the cell surface. C4-2 cells with 5% FBS on 2D (A) or in 3D HA hydrogel for 3 days (B) immunostained for RHAMM (green) and nuclei (blue). Arrows indicate differences in nuclear RHAMM between the two culture conditions. A quantification method was utilized that measured RHAMM staining intensity across a single line both inside and outside of the nucleus (C). Arrows indicate marked points to average staining intensity between and outside of as depicted in the chart to the right. Results of the quantification method show nuclear and cytoplasmic or membrane (cyto/mem)-bound RHAMM for both culture conditions (D). Western blotting for RHAMM expression after five days of 2D or 3D culture with β-actin used as a load control (E).

Next, we investigated the effects of 3D HA hydrogel culture on RHAMM expression and localization. As shown by immunofluorescence, C4-2 cells cultured on 2D (Figure 5A) have stronger nuclear RHAMM localization compared to cells cultured in 3D HA hydrogel (Figure 5B). To quantify this difference in localization, LSM imaging software was used to measure the pixel density of RHAMM staining in a single line across each cell’s width (Figure 5C). The average RHAMM pixel density was measured inside and outside the nucleus for each cell. The compiled results are shown in Figure 5D. In 3D HA hydrogel culture, nuclear RHAMM staining is less than 2D nuclear RHAMM staining. After five days of culture, RHAMM expression differences also were seen. C4-2 cultures in 2D showed less RHAMM expression when compared to those in 3D HA hydrogel culture (Figure 5E).

### RHAMM Knockdown

Because we found that the C4-2 cells were differentially expressing and localizing RHAMM in response to 3D HA hydrogel culture and that LNCaP cells expressed less RHAMM than C4-2 cells, we hypothesized that RHAMM expression was necessary for invadopodia and/or merging cluster formation in the HA hydrogel invasion assay. To test this hypothesis, we utilized a RHAMM knockdown approach on C4-2 cells.

#### RHAMM knockdown effects on 2D cell morphology

**Figure 6:**
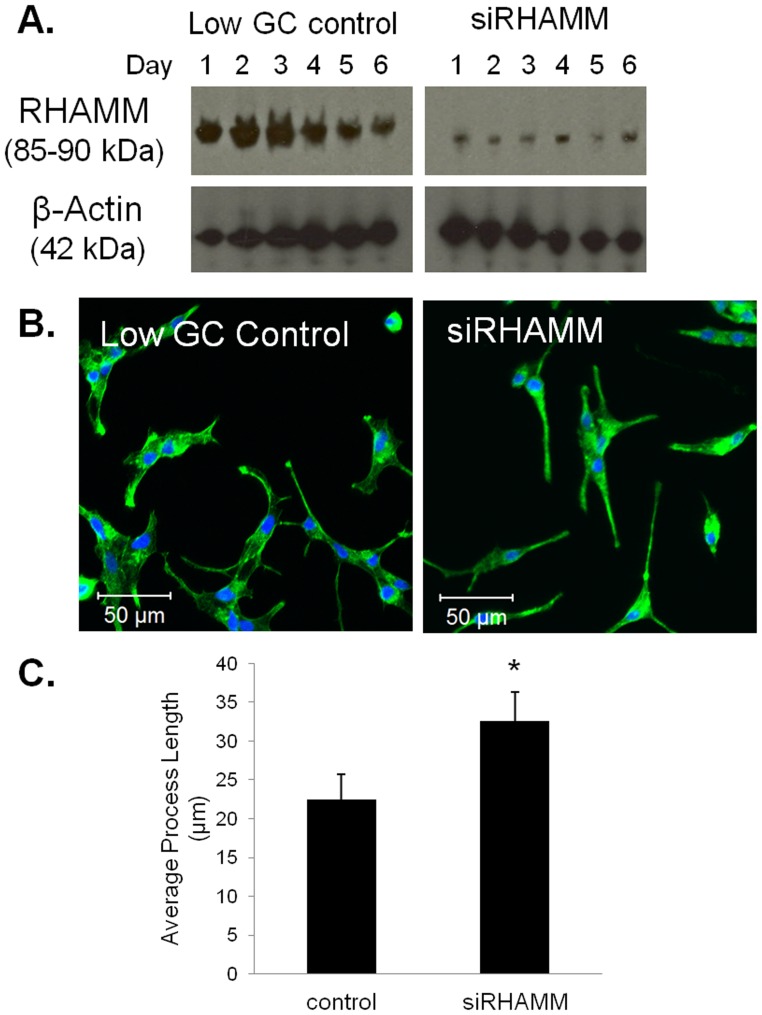
RHAMM knockdown C4-2 cells show longer cellular processes on 2D compared to control. RHAMM Western blotting for siRHAMM or low GC control transfected C4-2 cells across six days of culture with β-actin used as a load control (A). Fluorescent staining of siRHAMM or low GC control transfected cells at day 2 on 2D for F-actin (green) and nuclei (blue) (B). Quantification of average process length for all cellular processes within the imaged field (C). Error bars = SEM, n = 3, *p<0.05.

Figure 6A shows that our siRHAMM application was effective in lowering RHAMM expression in C4-2 cells compared to control. The two-day timepoint shows a 93% decrease in RHAMM expression, and the other timepoints show similar knockdown levels. The decrease in RHAMM expression is maintained for at least six days of culture. SiRHAMM treated C4-2 cells showed similar rates of growth compared to control cultures (data not shown).

When cultured on 2D and stained with phalloidin to visualize the cytoskeleton, siRHAMM C4-2 cells showed a different morphology compared to control cells (Figure 6B). SiRHAMM cells exhibited longer cellular processes compared to control (Figure 6C). The average number of processes per cell was unchanged between the two treatments (data not shown).

#### RHAMM knockdown effects on invadapodia and cluster formation in 3D

**Figure 7:**
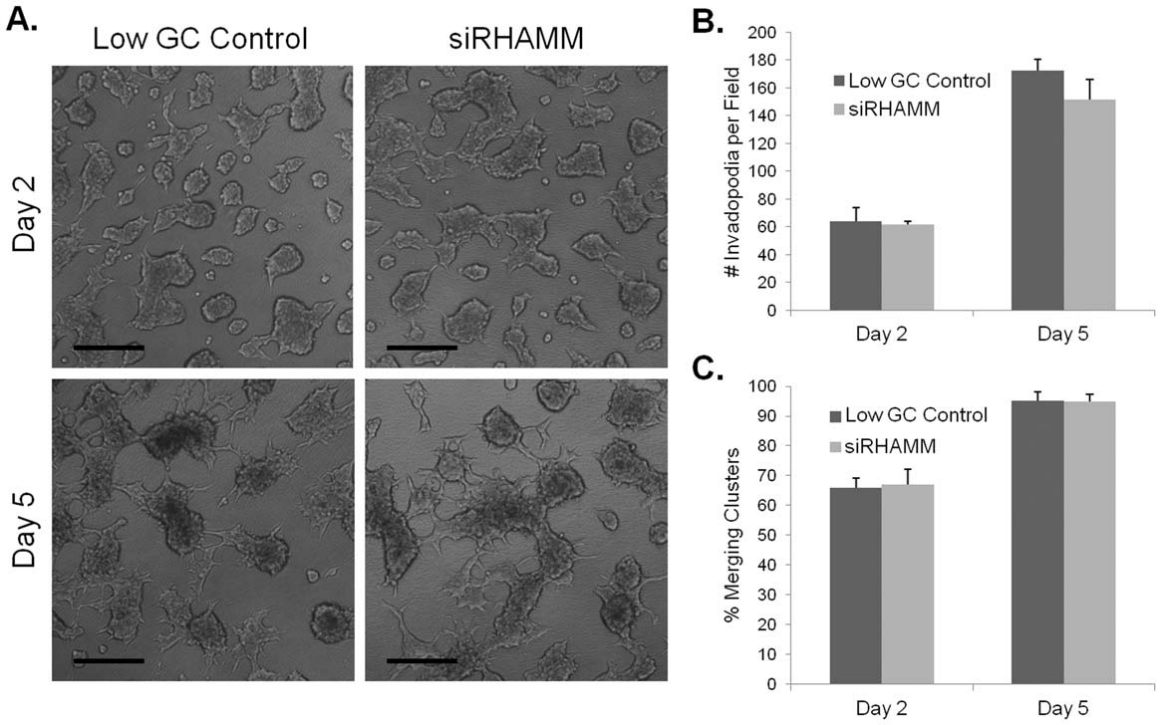
RHAMM knockdown C4-2 cells in 3D HA hydrogel invasion assay show no differences compared to control. Images of siRHAMM or low GC control transfected C4-2 cells cultured for two or five days with 5% FBS in the HA hydrogel invasion assay. Scale bars represent 200 µm (A). Quantification for average number of invadopodia (B) or percent merging clusters (C) in each imaged field. Error bars = SEM and n = 3.

To determine if the siRHAMM phenotype observed in 2D culture translated to differences in 3D culture, we cultured C4-2 cells in the 3D HA hydrogel invasion assay. Compared to control, siRHAMM treated cells showed no visible difference in morphology in the HA hydrogel invasion assay at either timepoint (Figure 7A). Both cultures showed large cell clusters with numerous processes as described above for C4-2 cells in 3D cultures. Quantification showed similar results. Both invadopodia number (Figure 7B) and merging cluster percentage (Figure 7C) were unchanged between siRHAMM and control cultures.

### HAase Expression in PCa Cells

**Figure 8:**
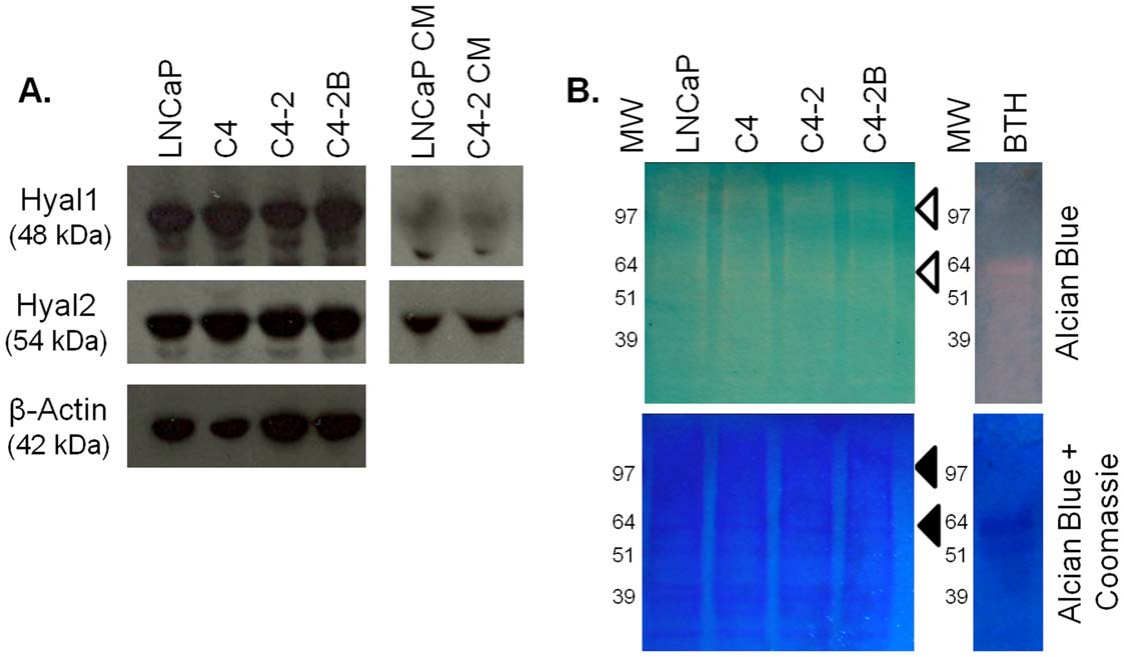
Cells of the LNCaP series show similar levels of HAase expression, secretion, and activity. Western blotting for Hyal1, Hyal2, and β-actin as a load control across the LNCaP cell series and for LNCaP and C4-2 conditioned medium (CM) (A). Hyaluronidase substrate gel activity assay for LNCaP series cell lysates and 50 µg BTH (B). Alcian blue stains HA (clear areas indicate HAase activity), Coomassie stain shows total protein as a load control. Clear arrows indicate major clear zones, black arrows indicate protein bands suitable for load controls, MW = molecular weight marker in kDa.

After determining that RHAMM knockdown did not affect invadopodia and cluster formation of C4-2 cells in the HA hydrogel invasion assay, we investigated if another family of HA interacting proteins, HAases, may be responsible. Western blot analysis showed that cells of the LNCaP series express both Hyal1 and Hyal2 (Figure 8A). Hyal2 was present in conditioned medium while Hyal1 was only weakly secreted from the cells. All cells of the series appeared to express and secrete similar levels of both HAases. To determine the activity of these HAases, a substrate gel assay was performed. All cells in the series showed HAase activity at similar levels as shown by the clear areas on the Alcian blue stained gel (Figure 8B). Coomassie blue staining for total protein confirmed that an equal amount of protein was added to each lane.

### Hyaluronidase Inhibition

**Figure 9:**
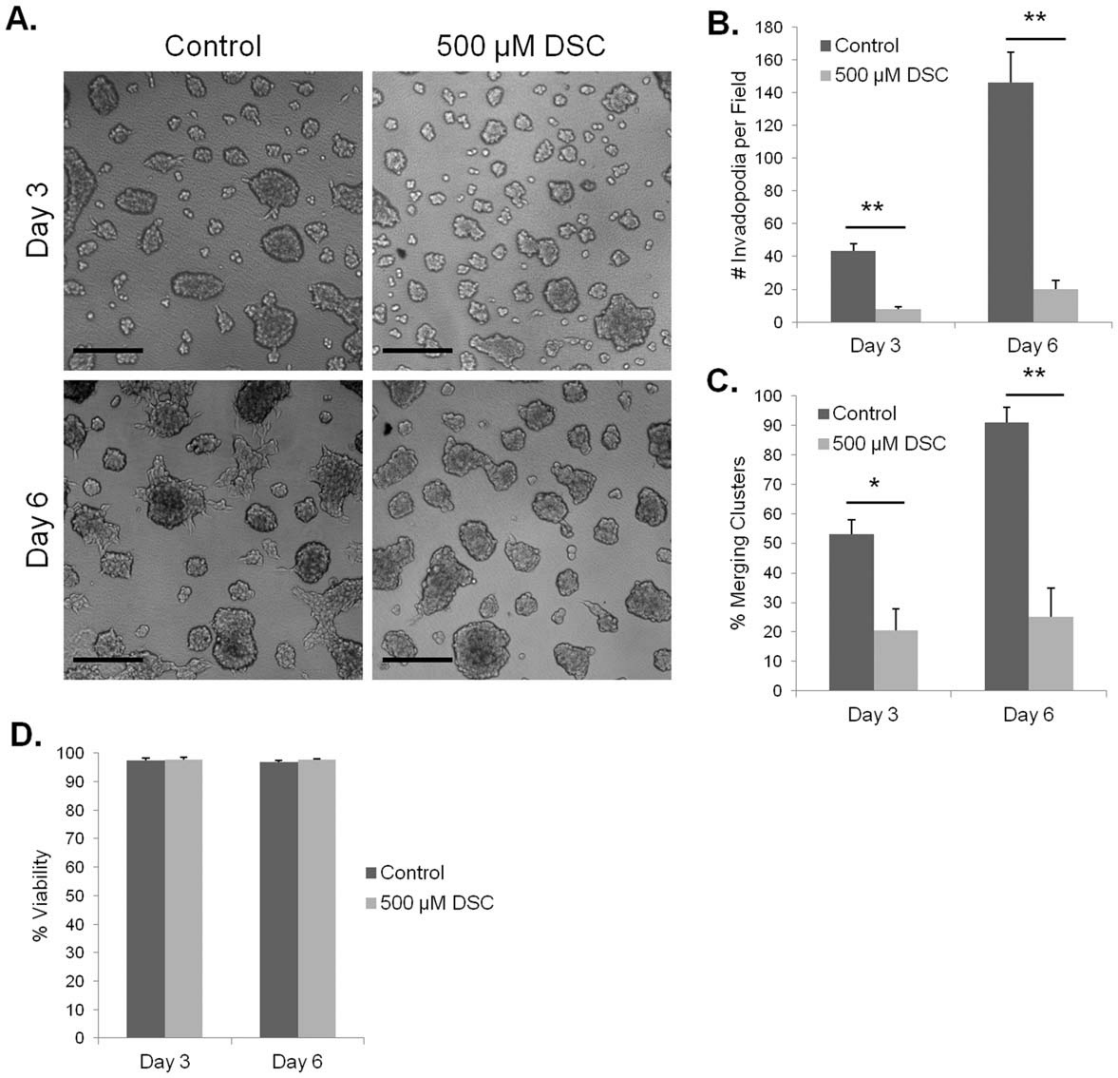
Invadopodia and cluster merging in the HA hydrogels depend on HAase activity. Images of C4-2 cells cultured for three or six days with 5% FBS and either 500 µM DSC or vehicle control in the HA hydrogel. Scale bars represent 200 µm (A). Quantification for average number of invadopodia (B) or percent merging clusters (C) in each imaged field. Trypan blue exclusion assay to test C4-2 cell viability at three and six days with either 500 µM DSC or vehicle control (D). Error bars = SEM, n = 3, *p<0.05, **p<0.01.

To determine if HAase expression was necessary for invadopodia and/or cluster formation in the HA hydrogel invasion assay, we utilized a HAase inhibitor, DSC. When applied to C4-2 cells in the HA hydrogel invasion assay at the IC-50 value of 500 µM, DSC showed noticeably smaller, unbranched cell clusters on day three compared to vehicle control, as well as a lack of invadopodia (Figure 9A). On day six a lack of invadopodia and cluster branching was still observed. Quantification showed a lower number of invadopodia in DSC-treated cultures at both three and six day timepoints (Figure 9B). Analysis of merging cluster percentages showed lower values for DSC treated cells at both timepoints as well (Figure 9C).

To ensure that the observed differences in invadopodia and merging cluster formation were not attributable to DSC toxicity to the cells, we performed a trypan blue exclusion assay. This assay tested C4-2 cell viability with 500 µM DSC at the three and six day timepoints used for the HA hydrogel invasion assay. The trypan blue assay showed similar levels of high cell viability (>95%) compared to vehicle control for cells observed at both timepoints (Figure 9D).

## Discussion

In this report, we provided data showing that the 3D HA hydrogel invasion assay provides an easily quantified, physiologically relevant method to study cancer invasion and the functions of individual HA interacting proteins likely to be involved in migration through HA-rich connective tissues. The quantifiable parameters of invadopodia and merging cluster formation were shown to be independent of cell number, and yielded low error values allowing for the use of statistical tests of significance. We suggest that this method could easily be modified to fit the unique experimental needs for examination of a variety of invasive cancer cells. While there is no doubt that there remain differences between soft, HA-rich connective tissues and the 3D HA hydrogels we use, this method offers a useful alternative to existing methods for mechanistic studies of cell invasion. As the field progresses, a deeper understanding of the mechanical properties and composition of these tissues will inform the development of more physiologically relevant hydrogels. Additionally, while understanding of how the chemically modified HA compares to native HA is somewhat limited, our data demonstrates that cells can interact with the crosslinked HA composing the hydrogel by degrading and binding it. How these interactions compare to interactions with native HA is unknown, but remains an exciting area of discovery.

Using this HA hydrogel method, we tested our hypothesis that the formation of invadopodia along with merging cluster formation that we observed in the HA hydrogels correlated with the ability of the PCa cells to interact with the hydrogel matrix by virtue of their expression of functional HA interacting proteins. Although it has been examined in several studies, the expression and function of the HA receptor, CD44, in prostate cancer invasion and in the LNCaP series of cell lines has failed to lead to general consensus. Some groups have reported a lack of CD44 mRNA and/or protein expression in these cell lines [39], [40] while others have found the receptor expressed as both mRNA and protein under certain conditions [41], [42]. Under the conditions wherein this work was performed, cells in 3D hydrogels actively moved through HA matrices while lacking expression of CD44, hence, other HA interacting proteins almost certainly were involved.

The alternative HA receptor, RHAMM/CD168, was an intriguing candidate because of its foundational role in cell motility through G protein dependent pathways [43], [44]. Unlike CD44, RHAMM was expressed in the LNCaP series cells growing in 3D hydrogels, and correlated with their ability to mimic prostate cancer progression. RHAMM expression levels were lower in LNCaP cells than the other, more aggressive and motile cells of the series. Interestingly, the expression of the higher molecular weight RHAMM isoform decreased as the cells became more aggressive, while the opposite was observed for the lower molecular weight isoform. This pattern has been associated previously with poor prognosis in multiple myeloma patients [45] and may be applicable for prostate cancer as well. If so, RHAMM isoform profiling may one day prove to be a useful new method of risk assessment for prostate cancer patients.

The RHAMM expression data, combined with the data showing lower amounts of invadopodia and merging cluster formation in LNCaP cells compared with C4-2, led us to wonder if RHAMM expression may be a driving factor behind motility in the LNCaP series. However, for RHAMM to interact with the HA hydrogel matrix, it would need to be on the cell surface, and RHAMM has been thought to be sequestered on the cell surface by binding to the surface receptor for HA, CD44 [11]. Without CD44 expression, it was unclear if RHAMM could still be localized on the cell surface. Using a cell surface biotinylation assay, we showed for the first time that RHAMM could be presented on the cell surface in the absence of CD44. This finding suggests many new questions. If not CD44, to what membrane moiety is RHAMM binding? Does cell surface RHAMM mediate different cellular functions when it is bound to CD44 in comparison to when it is free or bound to other moieties? Can RHAMM bind directly to the cell surface of some cancer cells? Further research beyond the scope of this study is necessary to answer these important questions.

To begin investigating if expression of RHAMM allows the C4-2 cells to functionally interact with the components of the HA hydrogel, we examined RHAMM expression and localization in 3D vs. 2D culture. We found that RHAMM was localized predominantly in the nucleus of cells grown in 2D, but a larger pool was found in the cytoplasm or membrane in 3D culture. This redistribution may reflect RHAMM translocalization from the nucleus to the cytoplasm or membrane in response to the HA present in the cells’ microenvironment or it may reflect a direct transport of newly synthesized RHAMM to the cell surface in cells grown in HA-rich 3D hydrogels. Supporting this, RHAMM expression in 3D culture was significantly higher (p = 0.013) than in 2D culture at day 5. These increases in RHAMM expression and cell surface and/or cytoplasmic localization also may lead to increased activation of downstream signaling pathways associated with motility [46]. Additionally, this data suggests that the chemical modifications did not compromise the biological activities of the HA comprising the hydrogel.

With the previous suggestion that RHAMM expression is related to PCa aggression [14], and our new data that PCa cells appear to be interacting with the hydrogel through changes in RHAMM expression and localization, we hypothesized that RHAMM expression was a driving factor behind invadopodia formation and the increases in numbers of merging clusters. To test this notion, we utilized siRNA knockdown methods. On 2D, siRHAMM knockdown C4-2 cells produced some long, straight cellular processes evident when compared to control transfectants. However, in 3D, siRHAMM C4-2 cells did not show any differences in numbers of invadopodia or in merging cluster formation when compared to control transfected cells. Therefore, the idea that RHAMM was a sole driving factor behind formation of cellular processes was not supported by the knockdown data. Thus, while our data does suggest that RHAMM expression plays a role in PCa progression and metastasis and perhaps in risk assessment, a decrease in RHAMM expression does not appear to prevent stromal invasion by C4-2 cells. For this reason, development of RHAMM targeted therapies seems unlikely to be useful for prevention of prostate cancer metastases.

Next, we investigated what role, if any, HAases play in PCa stromal invasion. HAases were expressed, secreted, and activated at similar levels at all stages of progression the cell lines represented. This suggests that even locally advanced prostate cancers, as represented by LNCaP cells, already have activated HAases that can assist in migration through HA-rich connective tissues. This data indicates that HA degradation by HAases may be as important in the development of early metastatic potential of a tumor as it is in the later stages of metastasis. Previous studies have shown that HAase secretion is 10 fold higher in PCa cells compared to both normal adult prostate and benign prostatic hyperplasia [47]. Therefore, along with HAase, other acquired properties affecting invadopodia and merging cluster formation must account for the differences that we observed between LNCaP and C4-2 cells grown in 3D hydrogels.

To test the need for active HAase in prostate cancer cell motility in 3D HA-rich hydrogels, we utilized a HAase inhibitor, DSC, and C4-2 cells treated at its IC-50 concentration of 500 µM [22], [37]. DSC treatment decreased both the number of invadopodia and the extent of cluster convergence seen in the HA hydrogels when compared to vehicle-treated controls. These results suggest that HAase activity is necessary, but not sufficient, for PCa cells to acquire the ability to invade HA-rich connective tissue stroma.

Invadopodia are formed through a combination of both production of degradative enzymes and activation of motility promoting proteins [48]. The degradative enzymes clear the matrix surrounding the cells and the motility promoting proteins, such as Rho GTPases, encourage actin reorganization allowing the cell to put forth the invadopodium [49]. We show that HAases can aid in the development of invadopodia through their role as a degradative enzyme. Interestingly, Na^2+^/H^+^ exchanger 1 (NHE1) is localized to invadopodia, acidifying the invadopodial space [50]. NHE1 activates hyaluronidases by lowering the pH to an optimal level [51]. Another contributing factor could be Rho GTPase activation. C4-2 cells have higher levels of Rac1-GTP compared to LNCaP cells [52]. Rac1 contributes to cell migration and invadopodia formation [49], [53].

Compelling arguments can be made suggesting Hyal1, Hyal2, or a combination of the two enzymes, act as driving force(s) behind PCa stromal invasion. Hyal1 has been most strongly implicated in the metastasis of several cancer types, including PCa [54]. Also, as an acid-active HAase, Hyal1 activation may explain the function NHE1 plays when localized to invadopodia [10], [50]. On the other hand, Hyal2 is found on the cell surface where it interacts with HA in the microenvironment. Additionally, Hyal2 interacts with HA receptors, also possibly contributing to stromal invasion [51]. Further research is needed to determine which HAase(s) is the principle player in PCa invasion and metastasis.

Taken together, our results suggest that DSC, and other HAase inhibitors, may be effective in preventing stromal invasion by PCa cells, even early in the course of locally invasive disease. In combination with other interventions, DSC or other HAase inhibitors, such as ascorbic acid derivatives or alkyl gallates, could be incorporated into treatment regima for PCa patients to help prevent tissue invasion and metastasis. DSC is currently FDA approved for asthma and allergy treatment, making it an attractive candidate for further pre-clinical study [24], [25].

### Conclusions

Here, we describe the development and use of a novel 3D HA hydrogel system that can be used to study aspects of invasion, including formation of invadopodia. This assay provides a more typical microenvironment for PCa cells that have metastasized to the bone marrow or other HA-rich connective tissues. Invasion in this assay can be quantified by counting the number of invadopodia and merging cell clusters. Invasion also depends on the ability of the PCa cells to interact with the HA within the hydrogel matrix. Knockdown of RHAMM expression did not affect invadopodia or merging cluster formation, while inhibition of HAase activity decreased both invadopodia and merging cluster numbers. HAase expression and activity are necessary but not sufficient for PCa cell invasion in the HA hydrogel. HAases may play an essential role in PCa invasion through HA-rich connective tissues, such as the bone marrow. Therefore, HAase inhibitors are attractive candidates for drugs to prevent PCa invasion and metastasis even early in disease progression.

## Supporting Information

Table S1 Mean Cell Counts of LNCaP and C4-2 Cells Growing in HA Hydrogels. Cell numbers were determined by counting cells in clusters as described in Materials and Methods.
(DOCX)

## References

[1] Society AC (2012) Cancer Facts and Figures 2012.

[2] Coleman RE (2012) Clinical Features of Metastatic Bone Disease and Risk of Skeletal Morbidity. Clinical Cancer Research 12: 6243s–6249s. 10.1158/1078-0432.CCR-06-0931 17062708

[3] Decaestecker C, Debeir O, Van Ham P, Kiss R (2007) Can anti-migratory drugs be screened in vitro? A review of 2D and 3D assays for the quantitative analysis of cell migration. Medicinal Research Reviews 27: 149–176. 16888756 10.1002/med.20078

[4] Yamaguchi H, Wyckoff J, Condeelis J (2005) Cell migration in tumors. Current Opinion in Cell Biology 17: 559–564. 16098726 10.1016/j.ceb.2005.08.002

[5] Marshall J (2011) Transwell() invasion assays. Methods in molecular biology (Clifton, NJ) 769: 97–110. 10.1007/978-1-61779-207-6_8 21748672

[6] van Horssen R, ten Hagen TLM (2011) Crossing Barriers: The New Dimension of 2D Cell Migration Assays. Journal of Cellular Physiology 226: 288–290. 20658519 10.1002/jcp.22330

[7] Clark BR, Keating A (1995) Biology of bone marrow stroma. Bone Marrow Transplantation: Foundations for the 21st Century 770: 70–78. 10.1111/j.1749-6632.1995.tb31044.x 8597383

[8] Schade UM, Nehmann N, Horny HP, Prehm P, Delpech B, et al (2006) Hyaluronate and its receptors in bone marrow. Acta Histochemica 108: 141–147. 16713618 10.1016/j.acthis.2006.03.018

[9] Meyer K (1958) CHEMICAL STRUCTURE OF HYALURONIC ACID. Federation Proceedings 17: 1075–1077. 13619775

[10] Stern R (2008) Hyaluronidases in cancer biology. Seminars in Cancer Biology 18: 275–280. 18485730 10.1016/j.semcancer.2008.03.017

[11] Hamilton SR, Fard SF, Paiwand FF, Tolg C, Veiseh M, et al (2007) The hyaluronan receptors CD44 and Rhamm (CD168) form complexes with ERK1,2 that sustain high basal motility in breast cancer cells. Journal of Biological Chemistry 282: 16667–16680. 17392272 10.1074/jbc.M702078200 PMC2949353

[12] Turley EA, Austen L, Vandeligt K, Clary C (1991) HYALURONAN AND A CELL-ASSOCIATED HYALURONAN BINDING-PROTEIN REGULATE THE LOCOMOTION OF RAS-TRANSFORMED CELLS. Journal of Cell Biology 112: 1041–1047. 1705559 10.1083/jcb.112.5.1041 PMC2288867

[13] Ekici S, Cerwinka WH, Duncan R, Gomez P, Civantos F, et al (2004) Comparison of the prognostic potential of hyaluronic acid, hyaluronidase (HYAL-1), CD44v6 and microvessel density for prostate cancer. International Journal of Cancer 112: 121–129. 15305383 10.1002/ijc.20368

[14] Gust KM, Hofer MD, Perner SR, Kim R, Chinnaiyan AM, et al (2009) RHAMM (CD168) Is Overexpressed at the Protein Level and May Constitute an Immunogenic Antigen in Advanced Prostate Cancer Disease. Neoplasia 11: 956–963. 19724689 10.1593/neo.09694 PMC2735808

[15] Bharadwaj AG, Kovar JL, Loughman E, Elowsky C, Oakley GG, et al (2009) Spontaneous Metastasis of Prostate Cancer Is Promoted by Excess Hyaluronan Synthesis and Processing. American Journal of Pathology 174: 1027–1036. 19218337 10.2353/ajpath.2009.080501 PMC2665762

[16] Gomez CS, Gomez P, Knapp J, Jorda M, Soloway MS, et al (2009) Hyaluronic Acid and HYAL-1 in Prostate Biopsy Specimens: Predictors of Biochemical Recurrence. Journal of Urology 182: 1350–1355. 19683287 10.1016/j.juro.2009.06.070 PMC2828051

[17] Hofinger ESA, Hoechstetter J, Oettl M, Bernhardt G, Buschauer A (2008) Isoenzyme-specific differences in the degradation of hyaluronic acid by mammalian-type hyaluronidases. Glycoconjugate Journal 25: 101–109. 17620008 10.1007/s10719-007-9058-8

[18] Harada H, Takahashi M (2007) CD44-dependent intracellular and extracellular catabolism of hyaluronic acid by hyaluronidase-1 and-2. Journal of Biological Chemistry 282: 5597–5607. 17170110 10.1074/jbc.M608358200

[19] Csoka AB, Frost GI, Stern R (2001) The six hyaluronidase-like genes in the human and mouse genomes. Matrix Biology 20: 499–508. 11731267 10.1016/s0945-053x(01)00172-x

[20] Huxley-Jones J, Foord SM, Barnes MR (2008) Drug discovery in the extracellular matrix. Drug Discovery Today 13: 685–694. 18583179 10.1016/j.drudis.2008.05.005

[21] Russ AP, Lampel S (2005) The druggable genome: an update. Drug Discovery Today 10: 1607–1610. 16376820 10.1016/S1359-6446(05)03666-4

[22] Kakegawa H, Matsumoto H, Satoh T (1985) ACTIVATION OF HYALURONIDASE BY METALLIC SALTS AND COMPOUND 48/80, AND INHIBITORY EFFECT OF ANTI-ALLERGIC AGENTS ON HYALURONIDASE. Chemical & Pharmaceutical Bulletin 33: 642–646. 3926329 10.1248/cpb.33.642

[23] Mio K, Stern R (2002) Inhibitors of the hyaluronidases. Matrix Biology 21: 31–37. 11827790 10.1016/s0945-053x(01)00185-8

[24] Chen JL, Moore N, Norman PS, Vanmetre TE (1969) DISODIUM CROMOGLYCATE A NEW COMPOUND FOR PREVENTION OF EXACERBATIONS OF ASTHMA. Journal of Allergy 43: 89–&. 10.1016/0021-8707(69)90129-4 4178832

[25] Edwards AM, Howell JBL (2000) The chromones: history, chemistry and clinical development. A tribute to the work of Dr R. E. C. Altounyan. Clinical and Experimental Allergy 30: 756–774. 10848895 10.1046/j.1365-2222.2000.00879.x

[26] Gurski LA, Jha AK, Zhang C, Jia XQ, Farach-Carson MC (2009) Hyaluronic acid-based hydrogels as 3D matrices for in vitro evaluation of chemotherapeutic drugs using poorly adherent prostate cancer cells. Biomaterials 30: 6076–6085. 19695694 10.1016/j.biomaterials.2009.07.054 PMC2782556

[27] Jia XQ, Colombo G, Padera R, Langer R, Kohane DS (2004) Prolongation of sciatic nerve blockade by in situ cross-linked hyaluronic acid. Biomaterials 25: 4797–4804. 15120526 10.1016/j.biomaterials.2003.12.012

[28] Wu TT, Sikes RA, Cui QJ, Thalmann GN, Kao CH, et al (1998) Establishing human prostate cancer cell xenografts in bone: Induction of osteoblastic reaction by prostate-specific antigen-producing tumors in athymic and SCID/bg mice using LNCaP and lineage-derived metastatic sublines. International Journal of Cancer 77: 887–894. 9714059 10.1002/(sici)1097-0215(19980911)77:6<887::aid-ijc15>3.0.co;2-z

[29] Thalmann GN, Sikes RA, Wu TT, Degeorges A, Chang SM, et al (2000) LNCaP progression model of human prostate cancer: Androgen-independence and osseous metastasis. Prostate 44: 91–103. 10881018 10.1002/1097-0045(20000701)44:2<91::aid-pros1>3.0.co;2-l

[30] Thalmann GN, Anezinis PE, Chang SM, Zhau HE, Kim EE, et al (1994) ANDROGEN-INDEPENDENT CANCER PROGRESSION AND BONE METASTASIS IN THE LNCAP MODEL OF HUMAN PROSTATE-CANCER. Cancer Research 54: 2577–2581. 8168083

[31] He XY, Sikes RA, Thomsen S, Chung LWK, Jacques SL (1994) PHOTODYNAMIC THERAPY WITH PHOTOFRIN-II INDUCES PROGRAMMED CELL-DEATH IN CARCINOMA CELL-LINES. Photochemistry and Photobiology 59: 468–473. 8022890 10.1111/j.1751-1097.1994.tb05066.x

[32] Thalmann GN, Sikes RA, Devoll RE, Kiefer JA, Markwalder R, et al (1999) Osteopontin: Possible role in prostate cancer progression. Clinical Cancer Research 5: 2271–2277. 10473115

[33] Zhang C, Soori M, Miles FL, Sikes RA, Carson DD, et al (2011) Paracrine Factors Produced by Bone Marrow Stromal Cells Induce Apoptosis and Neuroendocrine Differentiation in Prostate Cancer Cells. Prostate 71: 157–167. 20665531 10.1002/pros.21231 PMC2972389

[34] Sahiner N, Jha AK, Nguyen D, Jia XQ (2008) Fabrication and characterization of cross-linkable hydrogel particles based on hyaluronic acid: potential application in vocal fold regeneration. Journal of Biomaterials Science-Polymer Edition 19: 223–243. 18237494 10.1163/156856208783432462

[35] Guntenhoner MW, Pogrel MA, Stern R (1992) A SUBSTRATE-GEL ASSAY FOR HYALURONIDASE ACTIVITY. Matrix 12: 388–396. 1484506 10.1016/s0934-8832(11)80035-1

[36] Miura RO, Yamagata S, Miura Y, Harada T, Yamagata T (1995) ANALYSIS OF GLYCOSAMINOGLYCAN-DEGRADING ENZYMES BY SUBSTRATE GEL-ELECTROPHORESIS (ZYMOGRAPHY). Analytical Biochemistry 225: 333–340. 7762800 10.1006/abio.1995.1163

[37] Barla F, Higashijima H, Funai S, Sugimoto K, Harada N, et al (2009) Inhibitive Effects of Alkyl Gallates on Hyaluronidase and Collagenase. Bioscience Biotechnology and Biochemistry 73: 2335–2337. 10.1271/bbb.90365 19809167

[38] Shaw MT, MacKnight WJ (2005) Introduction to Polymer Viscoelasticity. Hoboken, NJ: John Wiley & Sons, Inc.

[39] Harrison GM, Davies G, Martin TA, Mason MD, Jiang WG (2006) The influence of CD44v3-v10 on adhesion, invasion and MMP-14 expression in prostate cancer cells. Oncology Reports 15: 199–206. 16328056

[40] Ricciardelli C, Russell DL, Ween MP, Mayne K, Suwiwat S, et al (2007) Formation of hyaluronan- and versican-rich pericellular matrix by prostate cancer cells promotes cell motility. Journal of Biological Chemistry 282: 10814–10825. 17293599 10.1074/jbc.M606991200

[41] Hurt EM, Kawasaki BT, Klarmann GJ, Thomas SB, Farrar WL (2008) CD44(+)CD24(–) prostate cells are early cancer progenitor/stem cells that provide a model for patients with poor prognosis. British Journal of Cancer 98: 756–765. 18268494 10.1038/sj.bjc.6604242 PMC2259168

[42] Xiao WW, Graham PH, Power CA, Hao JL, Kearsley JH, et al (2012) CD44 is a biomarker associated with human prostate cancer radiation sensitivity. Clinical & Experimental Metastasis 29: 1–9. 21953074 10.1007/s10585-011-9423-7

[43] Goueffic Y, Guilluy C, Guerin P, Patra P, Pacaud P, et al (2006) Hyaluronan induces vascular smooth muscle cell migration through RHAMM-mediated PI3K-dependent Rac activation. Cardiovascular Research 72: 339–348. 16934786 10.1016/j.cardiores.2006.07.017

[44] Zhang SW, Chang MCY, Zylka D, Turley S, Harrison R, et al (1998) The hyaluronan receptor RHAMM regulates extracellular-regulated kinase. Journal of Biological Chemistry 273: 11342–11348. 9556628 10.1074/jbc.273.18.11342

[45] Reiman T, Maxwell CA, Adamia S, Belch AR, Pilarski LM (2003) A RHAMM splice variant correlates with significantly reduced survival and heralds relapse in multiple myeloma. Blood 102: 680A–680A.

[46] Maxwell CA, McCarthy J, Turley E (2008) Cell-surface and mitotic-spindle RHAMM: moonlighting or dual oncogenic functions? Journal of Cell Science 121: 925–932. 18354082 10.1242/jcs.022038

[47] Lokeshwar VB, Lokeshwar BL, Pham HT, Block NL (1996) Association of elevated levels of hyaluronidase, a matrix-degrading enzyme, with prostate cancer progression. Cancer Research 56: 651–657. 8564986

[48] Murphy DA, Courtneidge SA (2011) The ‘ins’ and ‘outs’ of podosomes and invadopodia: characteristics, formation and function. Nature Reviews Molecular Cell Biology 12: 413–426. 21697900 10.1038/nrm3141 PMC3423958

[49] Harper K, Arsenault D, Boulay-Jean S, Lauzier A, Lucien F, et al (2010) Autotaxin Promotes Cancer Invasion via the Lysophosphatidic Acid Receptor 4: Participation of the Cyclic AMP/EPAC/Rac1 Signaling Pathway in Invadopodia Formation. Cancer Research 70: 4634–4643. 20484039 10.1158/0008-5472.CAN-09-3813

[50] Busco G, Cardone RA, Greco MR, Bellizzi A, Colella M, et al (2010) NHE1 promotes invadopodial ECM proteolysis through acidification of the peri-invadopodial space. Faseb Journal 24: 3903–3915. 20547664 10.1096/fj.09-149518

[51] Bourguignon LYW, Singleton PA, Diedrich F, Stern R, Gilad E (2004) CD44 interaction with Na+-H+ exchanger (NHE1) creates acidic microenvironments leading to hyaluronidase-2 and cathepsin B activation and breast tumor cell invasion. Journal of Biological Chemistry 279: 26991–27007. 15090545 10.1074/jbc.M311838200

[52] Kobayashi T, Inoue T, Shimizu Y, Terada N, Maeno A, et al (2010) Activation of Rac1 Is Closely Related to Androgen-Independent Cell Proliferation of Prostate Cancer Cells Both in Vitro and in Vivo. Molecular Endocrinology 24: 722–734. 20203103 10.1210/me.2009-0326 PMC5417531

[53] Sequeira L, Dubyk CW, Riesenberger TA, Cooper CR, van Golen KL (2008) Rho GTPases in PC-3 prostate cancer cell morphology, invasion and tumor cell diapedesis. Clinical & Experimental Metastasis 25: 569–579. 18461284 10.1007/s10585-008-9173-3

[54] Lokeshwar VB, Rubinowicz D, Schroeder GL, Forgacs E, Minna JD, et al (2001) Stromal and epithelial expression of tumor markers hyaluronic acid and HYAL1 hyaluronidase in prostate cancer. Journal of Biological Chemistry 276: 11922–11932. 11278412 10.1074/jbc.M008432200

