# Supplementary material for: Hyaluronan (HA) Interacting Proteins RHAMM and Hyaluronidase Impact Prostate Cancer Cell Behavior and Invadopodia Formation in 3D HA-Based Hydrogels
Source: PLoS One. 2012 Nov 16;7(11):e50075. doi: 10.1371/journal.pone.0050075 (PMC3500332; doi:10.1371/journal.pone.0050075)
Supplement: Table S1 — Mean Cell Counts of LNCaP and C4-2 Cells Growing in HA Hydrogels. Cell numbers were determined by counting cells in clusters as described in Materials and Methods. (DOCX) [file pone.0050075.s001.docx]

| **Cell Line** | **Growth Conditions** | **Day** | **Mean Cell Count/Field ± SEM** |
| --- | --- | --- | --- |
| LNCaP | 5% FBS | 3 | 2818 ± 591 |
| LNCaP | 5% FBS | 6 | 2958 ± 437 |
| C4-2 | 5% FBS | 3 | 3124 ± 337 |
| C4-2 | 2% TCM | 3 | 3336 ± 514 |
| C4-2 | 5% FBS | 6 | 3415 ± 274 |
| C4-2 | 2% TCM | 6 | 3271 ± 688 |

Table S1. Mean Cell Counts of LNCaP and C4-2 Cells Growing in HA Hydrogels. Cell numbers were determined by counting cells in clusters as described in Materials and Methods.
